# A Rare Case of Aerococcus urinae Mitral Valve Infective Endocarditis With Multiple Septic Emboli to the Brain

**DOI:** 10.7759/cureus.96475

**Published:** 2025-11-10

**Authors:** Saif M Srouji, Mohammed G Elhassan

**Affiliations:** 1 Internal Medicine, Saint Agnes Medical Center, Fresno, USA

**Keywords:** aerococcus infective endocarditis, aerococcus urinae, bacterial endocarditis, cardiovascular disease, infectious disease, infective endocarditis, mitral valve disease, mitral valve endocarditis, septic emboli, septic stroke

## Abstract

*Aerococcus urinae* is a rarely encountered Gram-positive bacterium that primarily causes urinary tract infections (UTIs), but in rare cases, it can result in bloodstream infections such as bacteremia, infective endocarditis (IE), and septic embolization. Despite its low virulence, *A. urinae* endocarditis carries high morbidity and mortality due to diagnostic challenges and delayed recognition. We report a case of *A. urinae* IE involving the native mitral valve, complicated by severe mitral regurgitation and multiple cerebral septic emboli. The patient’s course was marked by rapid clinical deterioration despite prompt initiation of guideline-directed antimicrobial therapy and multidisciplinary management involving cardiology, infectious disease (ID), intensive care, and cardiothoracic surgery teams. This case contributes to the growing body of literature recognizing *A. urinae* as a possible etiologic agent in elderly patients with urinary tract infections and systemic infection. Early echocardiographic assessment, microbial identification, and timely multidisciplinary management are essential to achieving favorable outcomes in such rare cases.

## Introduction

*Aerococcus urinae* is a catalase-negative, Gram-positive bacterium first described in the late 1980s as a cause of urinary tract infections (UTIs), particularly among elderly patients with underlying urologic abnormalities. Although often regarded as having low pathogenic potential, it has increasingly been implicated in invasive infections such as bacteremia and infective endocarditis (IE) [1,2]. Its morphological resemblance to staphylococci and streptococci on Gram stain frequently leads to misidentification and delays in appropriate antimicrobial therapy [1].

IE due to *A. urinae* is rare but notable for its high rate of systemic embolization, especially to the central nervous system (CNS), and its associated mortality [3,4]. Biofilm formation and strong valvular adherence may contribute to its embolic potential [3]. Early recognition requires a high index of suspicion, particularly in older adults with urinary tract disease or indwelling catheters who present with sepsis or new valvular dysfunction [3].

Diagnosis of IE is guided by the modified Duke criteria and echocardiographic assessment, with transesophageal echocardiography (TEE) offering superior sensitivity [5,6]. Management involves prolonged beta-lactam-based therapy, often in combination with an aminoglycoside, and surgical intervention may be warranted in complicated cases [5-11].

We present a fatal case of *A. urinae* IE of the native mitral valve, complicated by severe mitral regurgitation and multiple septic emboli to the brain. This case underscores the importance of early diagnosis, accurate organism identification, and multidisciplinary coordination to optimize outcomes in this uncommon but highly morbid infection.

## Case presentation

A 68-year-old female with a past medical history of primary hypertension, type 2 diabetes mellitus, mixed hyperlipidemia, chronic kidney disease stage IIIa, chronic obstructive pulmonary disease (COPD), and peripheral arterial disease presented to the emergency department (ED) due to nausea and vomiting in addition to fatigue of 10 hours duration. She was found at home covered in urine when emergency medical services arrived and was urgently transported to the ED. On presentation, she was slightly tachycardic and tachypneic; however, she was afebrile. Her vital signs are shown in Table 1.

**Table 1:** Initial patient vital signs on admission The patients initial vital signs upon presentation to the hospital. They are notable for tachycardia, hypertension, tachypnea and normal body temperature.

Parameter	Patient value	Reference range
Heart rate (HR)	113 beats/minute	60–100 beats/minute
Blood pressure (BP)	164/80 mmHg	<120/80 mmHg
Respiratory rate (RR)	34 breaths/minute	12–20 breaths/minute
Temperature (Celsius)	37.2 °C	36.1–37.5 °C

Upon further discussion with the patient, she mentioned that she also noticed she was having urgency and frequent urination. Physical examination noted an ill appearing female in respiratory distress, with severe lower limb pitting edema. No murmurs or abnormal heart sounds were noted. Due to her history of COPD and her current respiratory distress, a chest X-ray was obtained and revealed pulmonary edema. Thus, a subsequent transthoracic echocardiogram (TTE) was also ordered to measure left ventricular ejection fraction (LVEF) as there was suspicion of the presence of undiagnosed heart failure. She received one dose of furosemide 40 mg intravenously (IV).

Initial lab testing showed elevated lactate levels, leukocytosis, the presence of bacteria, and white blood cell casts in the urine. Findings are summarized in Table 2.

**Table 2:** Initial abnormal lab test results. Initial abnormal results of lab tests ordered on presentation to the hospital. The lab tests revealed elevated lactate levels, leukocytosis, and the presence of WBCs and RBCs in the urine, in addition to WBC casts, consistent with the diagnosis of a urinary tract infection.

Lab test	Patient results	Reference range
Serum lactate	2.2 mmol/L	0-2 mmol/L
White blood cells (WBCs)	13.7 K/mcL	4-11 K/mcL
Urine protein	30 mg/dL	<30 mg/dL
Urine white blood cells	>182/HPF	0-5 /HPF
Urine red blood cells	72/HPF	3-5/HPF
Urine blood	Moderate	Negative
Urine casts	WBC casts	None

Urine cultures did not show any growth initially, two sets of blood cultures were ordered, and she was started on ceftriaxone 1 G daily IV. The internal medicine service was consulted for inpatient admission to the medical floor for further management of severe sepsis stemming from a urinary tract infection.

**Figure 1:**
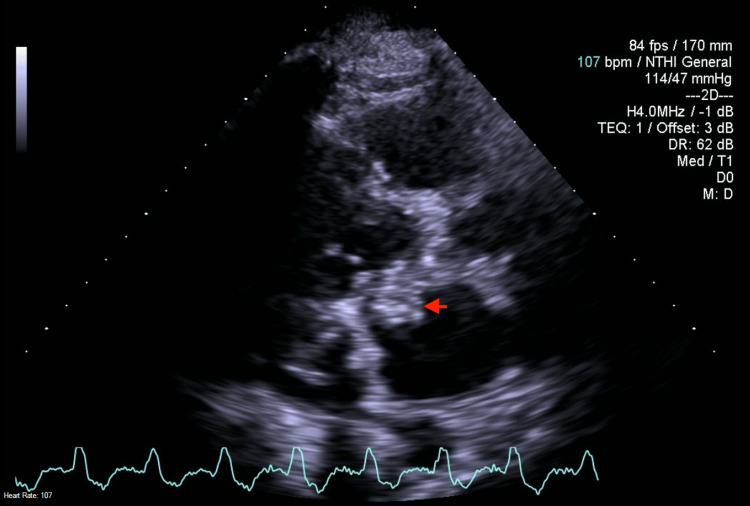
Transthoracic echocardiogram parasternal long view Transthoracic echocardiogram (TTE) in the parasternal long view showing a large vegetation (red arrow) on the anterior cusp of the mitral valve. This raised concerns for infective endocarditis and bacteremia in the setting of complicated urinary tract infection.

On day two of admission, the TTE was reported to show a large vegetation on the anterior leaflet of the mitral valve measuring approximately 10 mm in diameter, in addition to severe mitral regurgitation. The tricuspid valve was also reported to have moderate to severe regurgitation; however, the LVEF was 55-60%, which did not support a diagnosis of heart failure (Figure 1).

**Figure 2:**
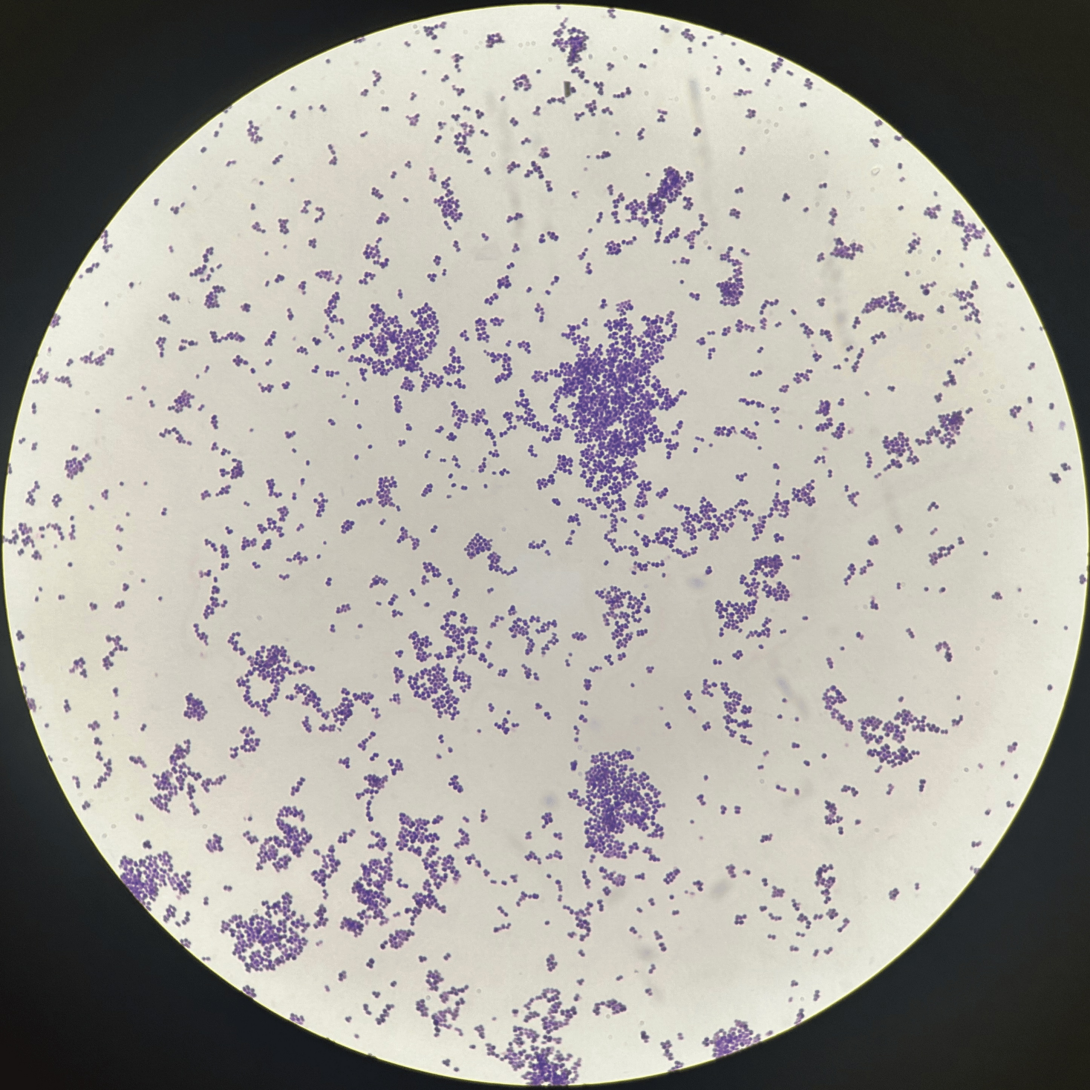
Blood cultures showing bacterial growth Blood cultures showing growth of Gram-positive bacteria in pairs and clusters that was confirmed to be *Aerococcus urinae*. In the setting of infective endocarditis, this bacteria was thought to be the pathogen.

**Figure 3:**
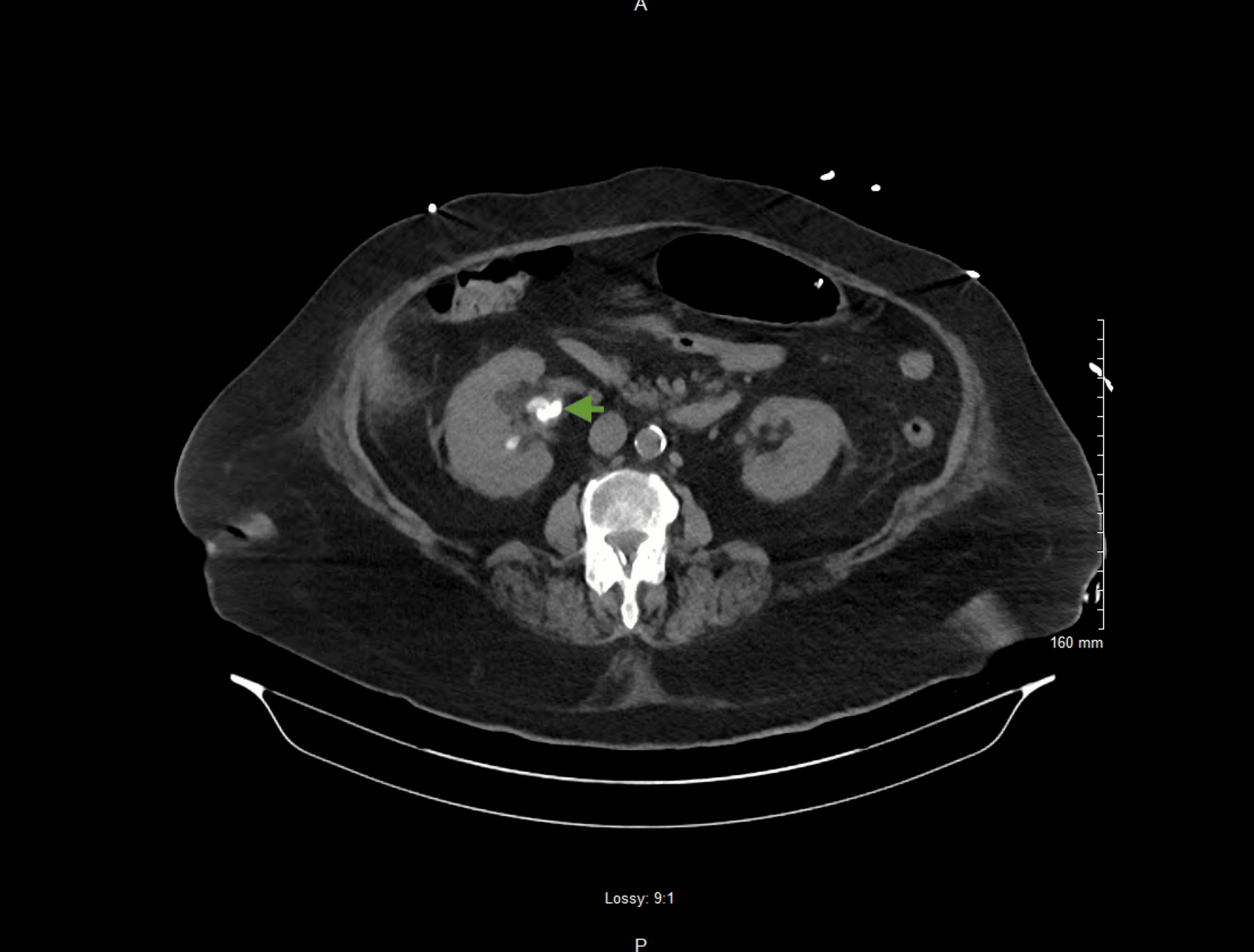
CT of the abdomen and pelvis revealing a kidney stone CT scan of the abdomen and pelvis without contrast, revealing a hyper density within the right renal pelvis (green arrow) measuring 37 mm x 17 mm in the largest diameter, which appears to be a staghorn calculus. This was possibly a risk factor for developing complicated urinary tract infection as there might have been bacterial film formation.

The medicine team continued treatment with IV ceftriaxone and added IV vancomycin for methicillin-resistant *Staphylococcus aureus* coverage, after initial Gram staining of the blood cultures showed growth of Gram-positive cocci in pairs and clusters, both in aerobic and anaerobic bottles within 48 hours of them being drawn (Figure 3). Throughout the day, she was noted to be having a lingering cough productive of white frothy sputum and remained tachypneic. Lung auscultation revealed crackles bilaterally, and SpO2 was 84% on room air. She was subsequently placed on bilevel positive airway pressure (BIPAP) with improvement in SpO2 to 97%. Later, her respiratory status somewhat improved and required O2 supplementation by nasal cannula only. The following day, the Cardiology team was consulted to weigh in on her condition and provide guidance on management, who, in turn, recommended consultation with ID. Now, at 72 hours after the drawing of blood cultures, they resulted in the growth of *A. urinae *(Figure 2). 

On day four of hospital admission, the ID team was consulted and recommended adjusting the antibiotic regimen to penicillin G 4 million units every four hours IV in addition to gentamicin 3 mg/kg/day IV (adjusted dose for renal dysfunction) per the bacterial culture sensitivity report (Table 3). The ID team also recommended consultation with the Cardiothoracic Surgery team. During the next couple of days, the Cardiothoracic Surgery team evaluated the patient and recommended that the patient be optimized from a medical standpoint, considering signs of acute-onset heart failure secondary to acute valvular dysfunction, as this is a high-risk surgery.

**Table 3:** Blood culture antimicrobial susceptibility report Antimicrobial susceptibility report for the blood cultures that showed growth of *A. urinae*. This result guided antimicrobial management.

Agent	Result	MIC
Ceftriaxone	Susceptible	0.25 mg/mL
Meropenem	Susceptible	0.06 mg/mL
Penicillin	Susceptible	0.06 mg/mL
Vancomycin	Susceptible	0.5 mg/mL

On the morning of the seventh day of hospital admission, she was noted to have encephalopathy and agitation secondary to respiratory distress, but was still able to protect her airway. Thus, she was transferred to the intensive care unit (ICU) and was placed on BIPAP again. Later, during the same day, she experienced sudden deterioration of mental status and became unresponsive to stimuli. A decision was made to intubate the patient and start mechanical ventilation for the protection of the airway and respiratory support. The ID team raised concerns about possible septic embolization of endocarditis vegetations and recommended brain imaging. A CT head without contrast was performed; however, it did not reveal any acute pathology, and a CT chest/abdomen/pelvis was most notable for the presence of hyperdensities within the right renal pelvis, raising concern for a staghorn calculus (Figure 3).

**Figure 4:**
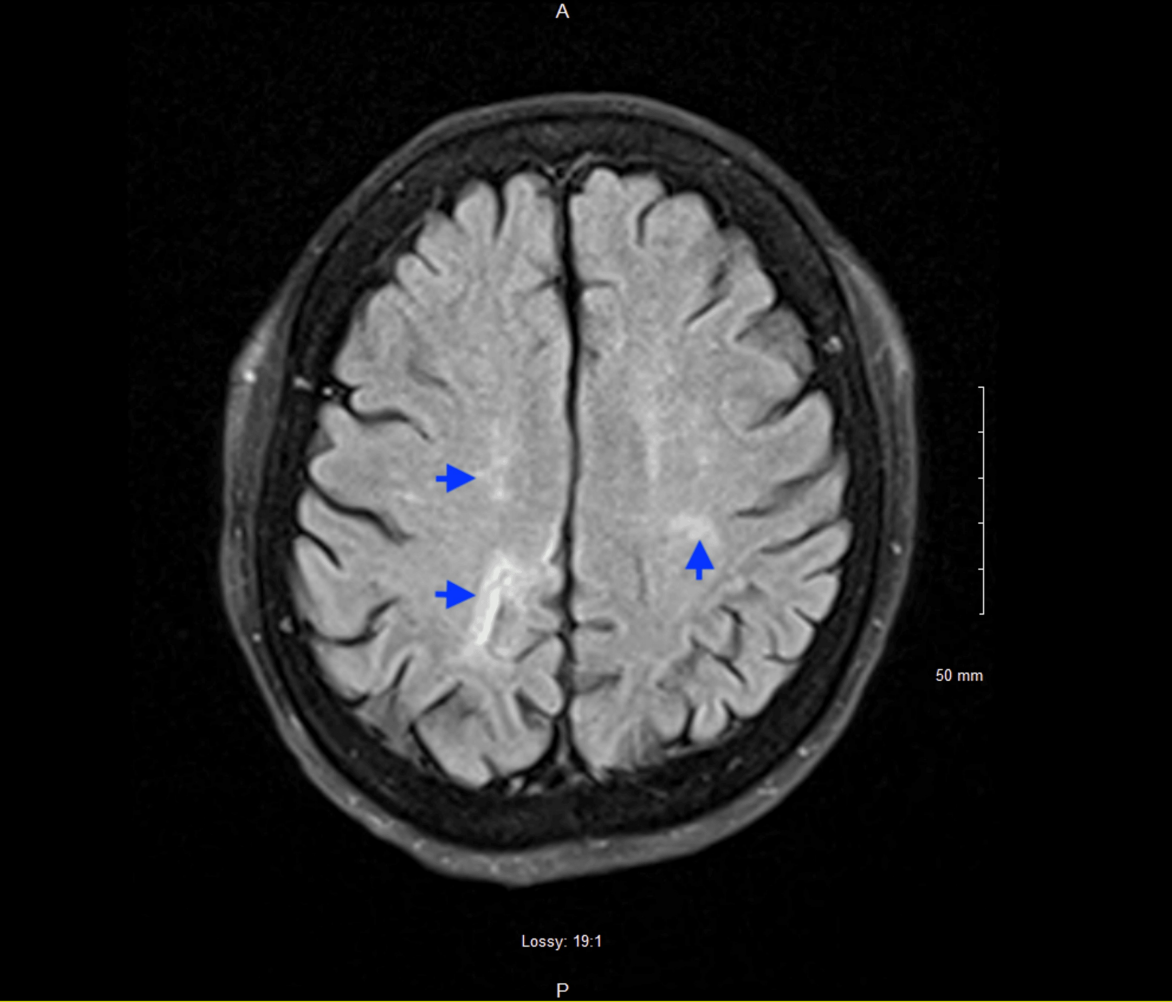
Brain MRI T2 sequence showing multiple infarcts Brain MRI obtained showing multiple hyperintensities (blue arrows) consistent with multiple septic emboli to the brain in the setting of bacteremia and infective endocarditis.

Brain MRI with and without contrast was ordered and showed multiple small subcentimeter foci of diffusion restriction in the right cerebellum and bilateral frontoparietal white matter, favored to represent acute infarcts. There were additional foci of diffusion hyperintensity in the right parietal lobe and right posterior corpus callosum, an area of faint diffusion hyperintensity with slight contrast enhancement along the posterior left frontal lobe, multiple punctate 3-4 mm foci of contrast enhancement along the left medial caudate head, and right medial thalamus without diffusion hyperintensity, all favored to represent subacute infarcts (Figure 4).

The following day, cold upper extremities were noted, raising concern for additional embolic phenomena. Over the next couple of days and upon detailed discussion between the ICU team, the Cardiology team, the Cardiovascular Surgery team, the ID team, the Palliative Care team, and the patient’s family, a mutual decision was made to continue medical therapy with IV antibiotics for the time being, while they work on transferring the patient to a higher level of care considering the complexity of this case due to the large size of mitral valve vegetation, acute onset of decompensated heart failure, concerning new developments of systemic septic embolization, in addition to the multiple comorbidities that the patient had.

**Figure 5:**
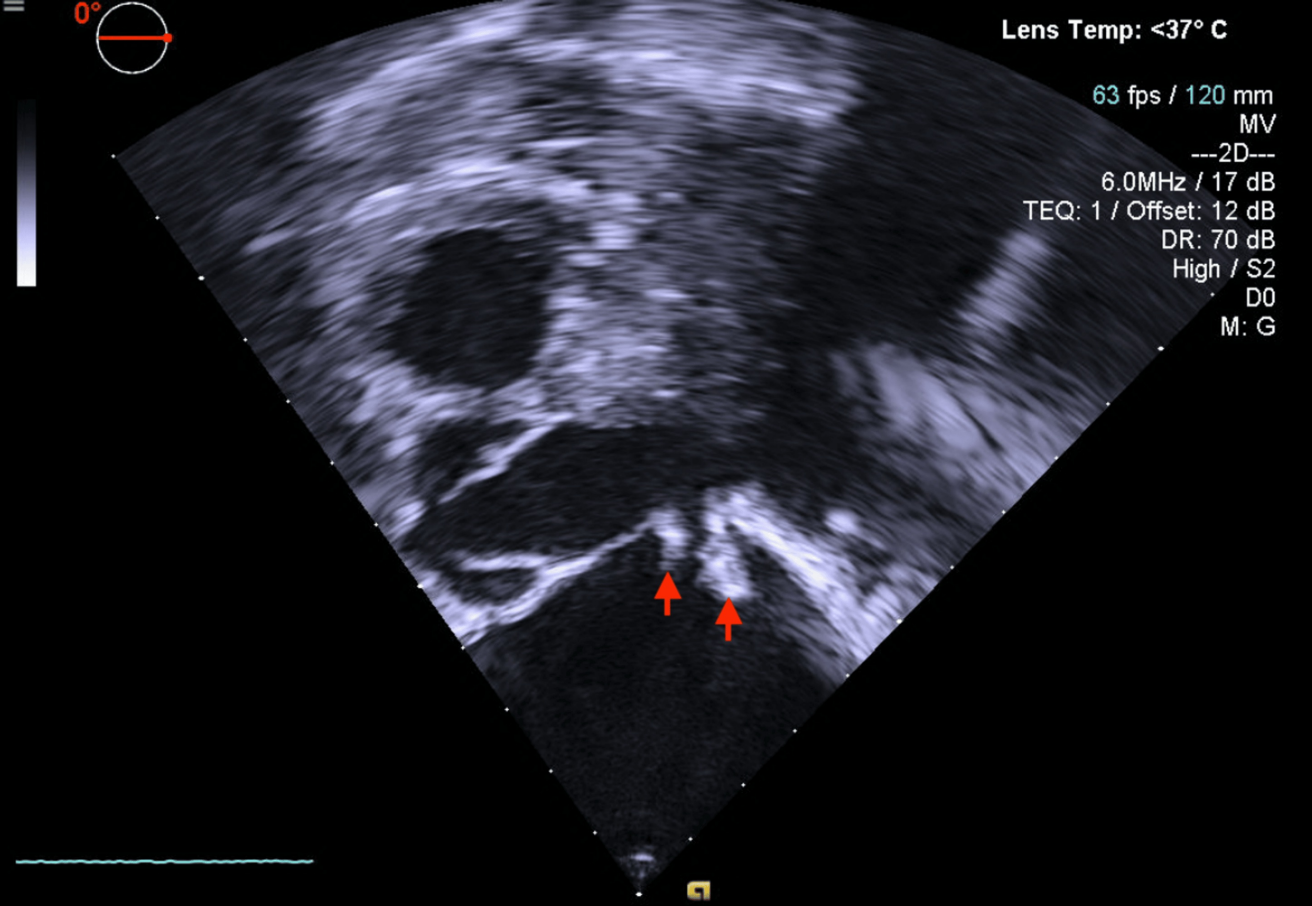
Transesophageal echocardiogram revealing vegetations A transesophageal echocardiogram revealing multiple vegetations (red arrows) on the cusps of the mitral valve confirming the diagnosis of infective endocarditis in the setting of bacteremia.

On day 10 of hospital admission, the sedation was weaned off, and the patient’s mental status recovered with major neurological deficits resulting from septic embolization to the CNS; however, it seemed that the patient had developed ICU-acquired weakness. Spontaneous breathing trial was attempted; however, it was aborted secondary to tachypnea, where RR was noted to be >45 breaths per minute, in addition to consistently low tidal volumes (TV), which were consistently <200 mL. Another detailed discussion regarding goals of care occurred after which the patient opted to change her code status to "Do Not Resuscitate" while still attempting all medical means of treatment. Because she continued to be febrile, a transesophageal echocardiogram (TEE) was obtained which redemonstrated the presence of a large vegetation on the anterior leaflet of the mitral valve with severe mitral valve regurgitation, however now also showed the presence of vegetation on the posterior leaflet of the mitral valve in addition to a small echodensity on the aortic valve concerning for vegetation as well (Figure 5).

On day 11 of hospital admission and upon review of the newly acquired TEE imaging, the Cardiothoracic Surgery team deemed the patient to be at extreme high risk for surgery, based on Society of Thoracic Surgeons (STS) scoring for operative risk, which noted an 11% risk of mortality and a 45% risk of morbidity. The following day, she was informed that she was accepted for transfer to a quaternary care center; however, she declined and opted for cessation of life-sustaining medical treatment, choosing to shift focus to a comfort-based care approach. At this stage, the patient was communicating her preferences with the Medical team through writing. On day 13 of hospital admission, she was extubated to a nasal cannula. Approximately 90 minutes later, she passed away.

## Discussion

*A. urinae* was first isolated from urine cultures in patients with UTIs, and the first report on this bacterium was published in 1989 [12]. It was first named in 1992 [13]. This bacterium is a Gram-positive organism, appearing as cocci growing in pairs and clusters and producing alpha hemolysis in blood agar. It is leucine aminopeptidase (LAP) positive and pyrrolidinyl aminopeptidase (PYR) negative. *A. urinae* can form biofilms that significantly contribute to its virulence. It is often mistaken for *Staphylococcus* on Gram staining of isolates. A key method to differentiate it from the *Staphylococcus* species is that it is catalase-negative [1]. Making this distinction is important because it will have implications for the treatment approach. *A. urinae* is usually sensitive to penicillins and fluoroquinolones and is often resistant to aminoglycosides and other agents such as trimethoprim/sulfamethoxazole, which are often used in the treatment of UTIs [2,9-11].

It is generally considered to be of low pathogenicity, and although not a very common cause of UTIs, the most common presentations for this bacterium occur as UTIs; however, in rare situations, it can cause severe bloodstream infections and can lead to IE. The incidence of *A. urinae* urinary tract and bloodstream infections is estimated to be 54 and three per 1,000,000 per year, respectively. The risk factors for infection that are usually implicated are old age and underlying urogenital tract conditions, including renal stones in our case [12]. Although not a commonly faced infection, in IE cases specifically, it is associated with unusually high rates of septic embolization, especially to the CNS, which can result in devastating morbidity and mortality [3]. To elaborate more, in cases of IE in general, rates of systemic septic embolization have been reported to be between 20% and 50% of cases [14]. While in cases where IE is due to *A. urinae*, some studies have reported the rate of systemic septic embolization to be on the higher end of the spectrum at 40-55% of cases, highlighting the importance of reporting such cases [4].

In relation to cases of IE, diagnosis can be aided by the use of modified Duke criteria, and in terms of imaging, an echocardiogram is the initial gold standard for evaluating the presence of vegetations [6]. TTE might not visualize any vegetation, especially if they are small in size. In cases where the TTE study is limited due to other factors such as body habitus, location of the vegetation, or operator experience, TEE should be pursued. TTE has a sensitivity for detecting vegetations of approximately 70% as opposed to more than 90% for TEE. Determination of the causative organism is obtained via blood cultures. Identifying the type of bacteria is imperative to drive sensitivity-based antimicrobial management [5].

According to the 2020 American College of Cardiology/American Heart Association practice guidelines and the 2015 European Society of Cardiology guidelines for the management of IE, it is recommended that treatment with antimicrobials be guided by organism speciation and specific antibiotic sensitivity [7,8]. It is wise to treat with broad-spectrum antimicrobial agents initially until blood culture results are finalized. As for indications for early surgical management (that which occurs during initial hospitalization and before completion of full therapeutic course of antibiotic) of IE, these include valvular dysfunction resulting in symptoms of heart failure, cases of left-sided IE caused by *S. aureus*, fungal organisms or other highly resistant organisms, cases complicated by heart block, annular or aortic abscesses, or destructive penetrating lesions, and cases of persistent infection as manifested by persistent bacteremia or fevers lasting more than five days after onset of appropriate antimicrobial therapy [7].

Because IE is a condition with a high septic cerebral embolism rate occurring in approximately 40% of cases and in-hospital mortality rates reaching up to 30%, it must be both aggressively investigated and treated [5,15]. Major risk factors for CNS septic embolization include delay or lack of appropriate antimicrobial therapy, vegetation size >10mm, presence of multiple vegetations, IE affecting the valves in the left side of the heart, and when *S. aureus *is the causative organism, among others [16]. In line with this, the 2023 European Society of Cardiology (ESC) guidelines emphasize the importance of managing IE within a dedicated multidisciplinary “endocarditis team,” involving cardiologists, cardiac imaging experts, cardiovascular surgeons, infectious disease specialists, and microbiologists, to ensure timely diagnosis, coordinated decision-making, and improved clinical outcomes [8].

In this case, specifically, we believe that it is worthwhile to recognize the quality of the interdisciplinary team approach that, despite the unfortunate outcome, was invaluable in providing accurate and timely diagnosis, prognostication, and treatment.

## Conclusions

*A. urinae* is a bacterium that is generally of low pathogenicity, typically causing urinary tract infections; however, it can also be implicated in invasive infections such as IE. Isolating *A. urinae* in blood cultures may prove to be a challenge, however it has a generally favorable antimicrobial sensitivity profile. Based on existing data and sensitivity profiles for this bacterium, an antimicrobial regimen consisting of a beta-lactam agent and an aminoglycoside is recommended.

While truly an uncommon occurrence, cases of IE due to *A. urinae* can produce devastating outcomes. Because there is a high risk of systemic embolization associated with *A. urinae* IE, rapid definitive intervention, most importantly source control, should be considered early in the course of the disease, to prevent catastrophic events such as in this case. Interdisciplinary medical team management may prove to be the best way to approach such cases especially if a large valve vegetation is detected or if complications such as septic embolization occur.
